# Diagnostic performance of a point shear wave elastography (pSWE) for hepatic fibrosis in patients with autoimmune liver disease

**DOI:** 10.1371/journal.pone.0212771

**Published:** 2019-03-11

**Authors:** Dong Won Park, Yoon Jin Lee, Won Chang, Ji Hoon Park, Kyoung Ho Lee, Young Hoon Kim, Nam Kyu Kang, Jung Wha Chung, Hee Yoon Jang, Soomin Ahn, Haeryoung Kim, Sook-Hyang Jeong, Jin-Wook Kim, Eun Sun Jang

**Affiliations:** 1 Department of Internal Medicine, Seoul National University Bundang Hospital, Seoul National University College of Medicine, Seongnam, Republic of Korea; 2 Department of Radiology, Seoul National University Bundang Hospital, Seoul National University College of Medicine, Seongnam, Republic of Korea; 3 Department of Pathology, Seoul National University Bundang Hospital, Seoul National University College of Medicine, Seongnam, Republic of Korea; 4 Department of Pathology, Seoul National University Hospital, Seoul National University College of Medicine, Seoul, Korea; Institute of Medical Research A Lanari-IDIM, University of Buenos Aires-National Council of Scientific and Technological Research (CONICET), ARGENTINA

## Abstract

**Background & aims:**

Elastography point quantification is a convenient method for measuring liver stiffness. It can be performed simultaneously with conventional ultrasonography. This study aimed to evaluate its diagnostic performance for assessing hepatic fibrosis in patients with autoimmune liver disease (AILD), including autoimmune hepatitis (AIH) and primary biliary cholangitis (PBC).

**Methods:**

The diagnostic performance of elastography point quantification (ElastPQ) was evaluated and compared with that of serum fibrosis markers, including the aspartate aminotransferase to platelet ratio index (APRI) and fibrosis-4 (FIB-4), using the receiver operating characteristics analysis with histologic evaluation as the reference standard.

**Results:**

In 49 AIH patients, sensitivity and specificity of ElastPQ were 93.6% and 44.4%, respectively, for significant fibrosis (≥ F2, cutoff 4.47 kPa), and 63.6% and 86.8% for cirrhosis (F4, cutoff 9.28 kPa). In 41 PBC patients, they were 81.8% and 73.3%, respectively, for significant fibrosis (≥ F2, cutoff 5.56 kPa), and 100% and 81.6%, respectively, for advanced fibrosis (≥ F3, cutoff 6.04 kPa). The areas under the receiver operating characteristic curves of ElastPQ for significant fibrosis (0.77, 95% CI 0.67–0.86) and cirrhosis (0.81, 95% CI 0.65–0.96) were higher than those of APRI and FIB-4 in AILD patients. According to the multivariable analysis, histological activity, steatosis, and body max index (BMI) were not significant factors that influenced the result of ElastPQ.

**Conclusions:**

ElastPQ exhibited better diagnostic performance–without the influence of confounding factors–for assessing hepatic fibrosis in AILD patients than serum fibrosis markers.

## Introduction

Hepatic fibrosis staging has been used as a prognostic factor by clinicians to measure clinical outcomes and as an index to establish therapeutic plans in patients with chronic liver disease (CLD) [1, 2]. Thus far, invasive liver biopsy is considered as the “gold standard” for assessing hepatic fibrosis stages and necroinflammatory activity [3].

There has been much effort to find non-invasive methods for assessing hepatic fibrosis to overcome the limitations of liver biopsy, which may include complications, non-representative sampling, and difficult-to-repeat processes [3–6]. Several serum fibrosis markers along with routine laboratory tests, including aminotransferase to platelet ratio index (APRI) and fibrosis-4 (FIB-4), have been proposed to be useful for evaluating hepatic fibrosis in CLD patients.

Currently, transient elastography (TE), along with serum fibrosis markers, is a widely accepted non-invasive technique for measuring liver stiffness [7, 8]. According to a previous meta-analysis that included 50 studies enrolling viral hepatitis patients, TE showed to have excellent diagnostic performance for significant fibrosis and liver cirrhosis with the mean area under the receiver operating characteristic curves (AUC) of 0.84 (95% CI 0.82–86) and 0.94 (95% CI 0.93–95), respectively [9]. Therefore, many clinical guidelines for managing CLD recommend using these non-invasive fibrosis measurement tools [10–12]. Nevertheless, there are some limitations to these non-invasive methods. The serum fibrosis marker has limited accuracy in predicting the intermediate grade of hepatic fibrosis, because the diagnostic performance of those markers was mostly validated to differentiate liver cirrhosis (F4 fibrosis) and is known to be highly affected by hepatocyte injury resulting in greater transaminase elevation than fibrosis [13]. The reproducibility and the diagnostic performance of TE are affected by obesity, steatosis, and necroinflammatory activity [14, 15].

Recently, several ultrasound-based elastography has been introduced to assess hepatic fibrosis which can be classified into shear wave speed techniques including TE, point shear wave elastography (pSWE) and shear ware speed imaging and strain/displacement techniques including strain elastography according to guidelines [16, 17]. Unlike TE, pSWE can be performed with conventional B-mode using a single probe without any extra-equipment [18, 19]. Thus, it allows for an evaluation of not only the hepatic parenchyma, but also the degree of hepatic fibrosis simultaneously [20, 21].

The cause of underlying liver disease has been revealed to influence the diagnostic performance of non-invasive methods, like TE and serum fibrosis marker [9, 22, 23]. The diagnostic performance of pSWE for assessing hepatic fibrosis has been relatively well studied in chronic viral hepatitis, but not in AILD [24]. According to a recent study evaluating patients with AILD, AUC was 0.85 (95% CI 0.77–0.91) and 0.86 (95% CI 0.78–0.92), and the optimal cut-off values were 9.7 kPa and 16.3 kPa for hepatic fibrosis stage ≥ F2 and F4, respectively [23]. Nonetheless, there has been no study evaluating the performance of pSWE and comparing it with serum fibrosis markers for assessing hepatic fibrosis in AILD, including AIH and PBC.

Therefore, this study aimed to investigate the diagnostic performance of elastography point quantification (ElastPQ) which is a pSWE method and compare it with serum fibrosis markers for predicting hepatic fibrosis in patients with biopsy-proven AIH and PBC.

## Materials and methods

### Patients

A total of 102 patients with AILD who underwent both ElastPQ and percutaneous liver biopsy on the same day at Seoul National University Bundang Hospital between May 2014 and May 2017 were included in this study. Patients with overlap syndrome–AIH and PBC simultaneously (n = 11)–and unreliable acquisition (success rate below 60%, at least 10 valid measurements, n = 1) were excluded [25]. Finally, 49 AIH and 41 PBC patients were included in this study. The diagnosis of AIH relies on the revised original scoring system of the International Autoimmune Hepatitis Group (IAIHG) [26]. PBC was diagnosed if two of the following criteria were met: (a) more than two times the upper limit of serum alkaline phosphatase (ALP) or more than five times the upper limit of gamma-glutamyl transpeptidase (GGT); (b) positive antimitochondrial antibody (>1:40); and (c) compatible liver histology [27]. Blood tests, including liver function tests, such as platelet count, aspartate and alanine aminotransferase (AST and ALT), serum bilirubin and albumin, GGT, ALP, international normalized ratio (INR), and immunoglobulin G (IgG), were performed on the admission day for liver biopsy.

This study was approved by the institutional review board of Seoul National University Bundang Hospital (No. B-1708-414-103), and the requirement for informed consent was waived because all blood tests, fibrosis measurement, and liver biopsy of this study were performed within routine clinical practice.

### Liver stiffness measurement

The technique called ‘ElastPQ’ (C5-1 probe, iU22 ultrasound system, Philips Healthcare, Bothell, WA, USA), which employs the point shear wave speed measurement using the acoustic radiation force impulse technique, was conducted by two board-certified abdominal radiologists (11 and 10 years of clinical experience, respectively) to evaluate hepatic fibrosis. Patients had fasted for at least 6 hours and were placed in supine position. To minimize liver motion, the measurement was performed in the right intercostal space with the right arm extended above the head during breath-holding. After selecting the best acoustic window by ultrasound examination, the region of interest (ROI) was placed in the area of the right hepatic parenchyma, perpendicular to and about 2cm below the liver capsule, avoiding the large hepatic vessel, bile duct, and rib shadows. The unit of calculated values recorded in the screen was in kilopascal (kPa). The measurement was repeated 10 times and it with ≥ 60% of success rate (the ratio of successful acquisition to total acquisitions) was considered as reliable. The median value was used to predict the degree of liver stiffness.

### Liver fibrosis

Percutaneous liver biopsy was performed under the guidance of ultrasonography, right after ElastPQ, using an 18-guage core biopsy needle. Liver biopsy specimens were fixed in formalin and paraffin embedded. Three-micron-thick sections were evaluated by hematoxylin and eosin, Masson's trichrome, reticulin, and Perl's iron stain. All biopsy specimens were analyzed by an experienced pathologist, who was blinded to the clinical results. Liver fibrosis and necroinflammatory activity were evaluated semiquantitatively in accordance with the METAVIR classification [28, 29]. Fibrosis was staged on a scale from 0 to 4 : F0 = no fibrosis; F1 = portal fibrosis without septa, mild; F2 = portal fibrosis and few septa, significant; F3 = numerous septa without cirrhosis, advanced; F4 = cirrhosis. Significant fibrosis was defined as F2 or greater (≥ F2). Activity was graded as follows: A0 = none; A1 = mild; A2 = moderate; and A3 = severe.

### Serum biochemical marker assays

The parameters allowing the calculation of APRI and FIB-4 were determined using the blood test results from the admission day for liver biopsy. APRI and FIB-4 were calculated using the following formulas: (AST [IU/L] / upper normal limit of AST [IU/L]) / platelets [10^3^/mm^3^] and (age [years] × AST [IU/L]) / (platelets [10^3^/mm^3^] × ALT [IU/L]^1/2^), respectively [30, 31]. The diagnostic performance of APRI and FIB-4 in the assessment of hepatic fibrosis was compared with that of ElastPQ.

### Statistical analysis

Patient characteristics are provided as the mean ± standard deviation or as the median and interquartile range as appropriate for continuous variables. The trend between the elasticity values of ElastPQ and the stages of hepatic fibrosis was estimated using the Spearman’s rho coefficient. The diagnostic performance of ElastPQ for hepatic fibrosis was determined in terms of sensitivity, specificity, positive and negative predictive values, as well as likelihood ratio by receiver operating characteristic (ROC) curves. The optimal cut-off values between the stages of hepatic fibrosis were determined at the maximized sensitivity and specificity using the Youden’s index. The diagnostic performance of ElastPQ and serum biochemical markers (either of APRI or FIB-4) were compared by AUC using the method proposed by DeLong et al [32]. Standardization was also performed to minimize spectrum bias using the DANA method (differences between the mean advanced fibrosis stage and the mean non-advanced fibrosis stage) [33, 34]. Logistic regression analysis was carried out to evaluate the confounding variables affecting the performance of ElastPQ. All tests were two-sided with a significance level of 0.05. However, the results calculated using the Bonferroni correction to account for the multiple comparisons were considered as significant if *P*<0.05/2 (*P*<0.025). All statistical analyses were performed using SPSS version 22.0 (IBM Corp., NY, USA) and Stata version 14.0 (College Station, TX, USA).

## Results

### Patient characteristics

The characteristics of 90 AILD patients (49 AIH and 41 PBC) are summarized in Table 1. The scores of revised IAIHG criteria were ≥ 10 in all patients (mean = 15.8), which indicated ‘probable AIH’ at minimum. All patients with PBC satisfied the definite diagnostic criteria. Among the 90 patients, 20 patients (22.2%) were in METAVIR stage F0, 28 patients (31.1%) in stage F1, 19 patients (21.1%) in stage F2, 11 patients (12.2%) in stage F3, and 12 patients (13.3%) in stage F4. As for histological activity, 11 patients (12.2%) were in grade A0, 44 patients (48.9%) in grade A1, 19 patients (21.1%) in grade A2, and 16 patients (17.8%) in grade A3.

**Table 1:** Baseline characteristics of the patients.

	AIH (n = 49)	PBC (n = 41)	Total AILD (n = 90)
Sex, female*	42 (85.7)	35 (85.4)	77 (85.6)
Age, years old ^¶^	56.0 ± 15.5	55.3 ± 12.1	55.7 ± 14.0
BMI, kg/m^2^	23.7 (21.3–25.4)	25.5 (23.2–28.4)	24.9 (22.7–27.4)
Laboratory finding			
Platelet, 10^3^/mm^3^	197 (137–253)	227 (184–267)	216 (159–256)
AST, IU/L	97 (54–381)	40 (33–75)	68 (36–191)
ALT, IU/L	163 (53–563)	45 (29–78)	62 (38–232)
ALT < x5 ULN	28 (57.1%)	39 (95.1%)	67 (74.4%)
Bilirubin, mg/dL	1.3 (0.8–1.9)	0.6 (0.5–0.9)	0.8 (0.6–1.4)
GGT, IU/L	109 (69–187)	215 (99–444)	137 (85–275)
ALP, IU/L	133 (100–158)	187 (109–317)	149 (105–225)
Cholesterol, mg/dL	169 (147–193)	185 (164–223)	176 (152–202)
Albumin, g/dL	4.1 (3.7–4.3)	4.2 (4.0–4.3)	4.1 (3.8–4.3)
PT, INR	1.07 (1.02–1.16)	0.98 (0.95–1.02)	1.03 (0.96–1.06)
Clinical cirrhosis*	4 (8.2)	0 (0)	4 (8.2)
Steatosis (>33%)*	8 (16.3)	4 (9.8)	12 (13.3)
Fibrosis stage*			
F0	12 (24.5)	8 (19.5)	20 (22.2)
F1	6 (12.2)	22 (53.7)	28 (31.1)
F2	10 (20.4)	8 (19.5)	19 (21.1)
F3	10 (20.4)	2 (4.9)	11 (12.2)
F4	11 (22.4)	1 (2.4)	12 (13.3)
Activity grade*			
A0	3 (6.1)	8 (19.5)	11 (12.2)
A1	15 (30.6)	28 (68.3)	44 (48.9)
A2	16 (32.7)	4 (9.8)	19 (21.1)
A3	15 (30.6)	1 (2.4)	16 (17.8)

Data are median (interquartile range)

* N (%)

^¶^ mean ± standard deviation AIH, autoimmune hepatitis; PBC, primary biliary cholangitis; AILD, autoimmune liver disease; BMI, body max index; AST, aspartate aminotransferase; ALT; alanine aminotransferase; GGT, gamma-glutamyl transpeptidase; ALP, alkaline phosphatase; PT, prothrombin time

### Prediction of hepatic fibrosis by ElastPQ

**Fig 1:**
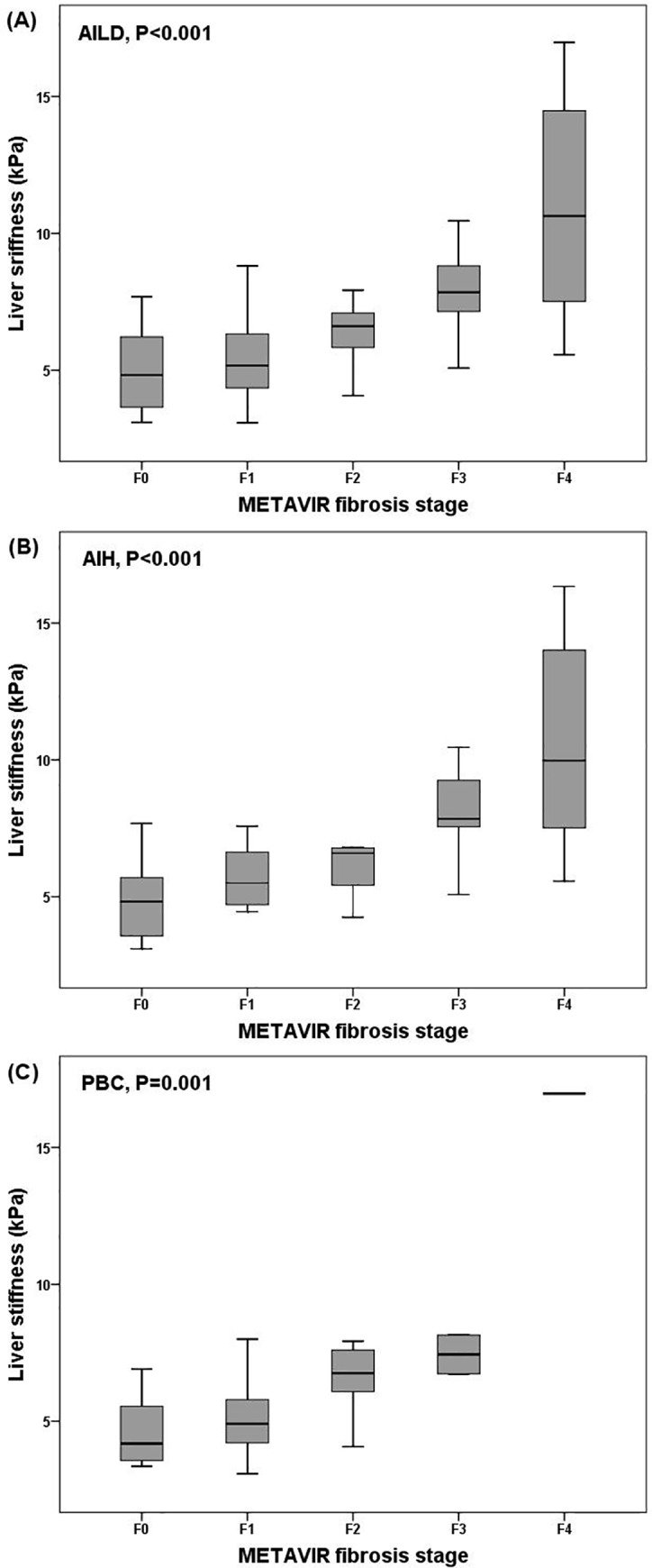
Box plots of elasticity values according to METAVIR hepatic fibrosis stage in patients with autoimmune liver disease (AILD) (A), autoimmune hepatitis (AIH) (B), and primary biliary cholangitis (PBC) (C). The Liver stiffness value was positively correlated with the stage of hepatic fibrosis.

The distribution of liver stiffness values according to the fibrosis stages in patients with AIH, PBC, or both (AILD) is shown in Fig 1. The values of ElastPQ in patients with AILD ranged from 3.08 to 16.97 kPa. Liver stiffness was positively correlated with the stages of hepatic fibrosis in patients with AILD (ρ = 0.53, *P*<0.001): AIH (ρ = 0.44, *P* = 0.002) and PBC (ρ = 0.62, *P*<0.001), respectively.

The overall diagnostic performance of ElastPQ for assessing hepatic fibrosis is summarized in Table 2. In patients with AILD, AUC for the prediction of significant fibrosis (≥ F2) was 0.77 (95% CI 0.67–0.85). Sensitivity and specificity of ElastPQ with the optimal cut-off value of 5.70 kPa to detect significant fibrosis (≥ F2) were 73.8% and 68.8%, respectively. AUC for the prediction of cirrhosis (F4) was 0.81 (95% CI 0.71–0.89). Sensitivity was 66.7% with the optimal cut-off value of 9.28 kPa; specificity was 92.3%. In patients with AIH, AUC (95% CI) was 0.70 (0.56–0.83) and 0.75 (0.61–0.87), and the optimal cut-off values were 4.47 kPa, and 9.28 kPa for hepatic fibrosis stage ≥ F2 and F4, respectively. In patients with PBC, AUC (95% CI) was 0.81 (0.65–0.91) and 0.91 (0.78–0.98), and the optimal cut-off values were 5.56 kPa and 6.04 kPa for significant fibrosis (≥ F2) and advanced fibrosis (≥ F3), respectively. After adjustment with the DANA method, some values were higher than the observed AUC (S1 Table). Both observed AUC (0.91, 95% CI 0.78–0.98) and adjusted AUC (0.93) for advanced fibrosis (≥ F3, n = 3) were highest in the PBC group. There was only one PBC patient in the stage of cirrhosis, so the cut-off value for cirrhosis (F4) was not determined.

**Table 2:** Diagnostic performance of ElastPQ for hepatic fibrosis stage.

	Cut-off (kPa)	AUC	Sensitivity (%)	Specificity (%)	PPV (%)	NPV (%)	LR +	LR -
**AILD**								
≥ F2	5.70	0.77 (0.67–0.85)	73.8 (58.0–86.1)	68.8 (53.7–81.3)	67.4 (52.0–80.5)	75.0 (59.7–86.8)	2.36 (1.5–3.7)	0.38 (0.2–0.7)
≥ F3	6.40	0.81 (0.71–0.88)	75.0 (53.3–90.2)	75.8 (63.6–85.5)	52.9 (35.1–70.2)	89.3 (78.1–96.0)	3.09 (1.9–5.0)	0.33 (0.2–0.7)
F4	9.28	0.81 (0.71–0.89)	66.7 (34.9–90.1)	92.3 (84.0–97.1)	57.1 (28.9–82.3)	94.7 (87.1–98.5)	8.67 (3.6–20.6)	0.36 (0.2–0.8)
**AIH**								
≥ F2	4.47	0.70 (0.56–0.83)	93.6 (78.6–99.2)	44.4 (21.5–69.2)	74.4 (57.9–87.0)	80.0 (44.4–97.5)	1.68 (1.1–2.6)	0.15 (0.03–0.6)
≥ F3	7.11	0.76 (0.62–0.87)	66.7 (43.0–85.4)	78.6 (59.0–91.7)	70.0 (45.7–88.1)	75.9 (56.5–89.7)	3.11 (1.4–6.7)	0.42 (0.2–0.8)
F4	9.28	0.75 (0.61–0.87)	63.6 (30.8–89.1)	86.8 (71.9–95.6)	58.3 (27.7–84.8)	89.2 (74.6–97.0)	4.84 (1.9–12.3)	0.42 (0.2–0.9)
**PBC**								
≥ F2	5.56	0.81 (0.65–0.91)	81.8 (48.2–97.7)	73.3 (54.1–87.7)	52.9 (27.8–77.0)	91.7 (73.0–99.0)	3.07 (1.6–5.9)	0.25 (0.07–0.9)
≥ F3	6.04	0.91 (0.78–0.98)	100 (29.2–100.0)	81.58 (65.7–92.3)	30.0 (6.7–65.2)	100 (88.8–100)	5.43 (2.8–10.6)	0

Data are expressed with 95% confidence interval

ElastPQ, elastography point quantification; kPa, kilopascal; AUC, area under the receiver-operator-characteristic curve; CI, confidence interval; PPV, positive predictive value; NPV, negative predictive value; LR+, positive diagnostic likelihood ratio; LR-,negative diagnostic likelihood ratio; AILD, autoimmune liver disease; AIH, autoimmune hepatitis; PBC, primary biliary cholangitis

### Parameters affecting the performance of ElastPQ

Univariable regression analysis of parameters affecting ElastPQ values was performed in patients with AIH and PBC, respectively. As shown in Table 3, variables associated with the value of ElastPQ were the serum ALT level (*P* = 0.042), histologic activity (P<0.001), and histologic hepatic fibrosis (P<0.001) in AILD. Moreover, male gender (*P* = 0.036) and histologic hepatic fibrosis (*P* = 0.002) were associated with ElastPQ in AIH, and histologic activity (*P* = 0.002) and histologic hepatic fibrosis (*P*<0.001) in PBC, respectively. According to the multivariable analysis, histologic fibrosis grade was the only variable affecting the value of ElastPQ in both AIH (*P* = 0.006) and PBC (*P* = 0.001) patients. Notably, histological activity (odds ratio [OR] 1.72; 95% CI 0.72–4.11; *P* = 0.215 and OR 2.19; 95% CI 0.73–6.56; *P* = 0.156) and steatosis grade (OR 1.27; 95% CI 0.14–11.77; *P* = 0.832 and OR 0.79; 95% CI 0.06–10.06; *P* = 0.852) were not independent contributing factors for elasticity measured by ElastPQ in both AIH and PBC patients.

**Table 3:** Regression analysis of the determinants for median ElastPQ value.

	Univariable		Multivariable	
Parameters	Exp (β) (95% CI)	P value	Exp (β) (95% CI)	P value
**Total AILD**				
Age, years old	0.99 (0.95–1.04)	0.838		
Male	3.74 (0.68–20.58)	0.128	1.51 (0.33–6.86)	0.593
BMI, kg/m^2^ ≥ BMI 25	0.94 (0.79–1.10) 1.30 (0.36–4.71)	0.429 0.689		
	
ALT, log IU/L **≥** UNL x 5	1.26 (0.79–2.03) 0.34 (1.04–1.74)	0.042 0.624	1.22 (0.81–1.85)	0.330
	
ALP, log IU/L	1.75 (0.66–4.64)	0.258		
GGT, log IU/L	1.14 (0.60–2.18)	0.684		
Steatosis	1.51 (0.28–8.05)	0.627		
Activity grade	3.09 (1.67–5.72)	<0.001	1.98 (1.11–3.53)	0.022
Fibrosis grade	3.19 (2.15–4.69)	<0.001	2.73 (1.81–4.13)	<0.001
**AIH**				
Age, years old	0.99 (0.94–1.05)	0.742		
Male	13.8 (1.19–139.7)	0.036	4.74 (0.39–58.33)	0.218
BMI, kg/m^2^	1.09 (0.84–1.42)	0.505		
≥ BMI 25	1.59 (0.25–10.19)	0.720		
ALT, log IU/L	1.09 (0.57–2.09)	0.779	1.44 (0.79–2.62)	0.222
**≥** UNL x 5	-0.36 (-2.17–1.45)	0.690		
IgG	1.01 (0.99–1.01)	0.360		
Steatosis	1.27 (0.14–11.77)	0.832		
Activity grade	1.85 (0.71–4.84)	0.204	1.72 (0.72–4.11)	0.215
Fibrosis grade	2.48 (1.44–4.27)	0.002	2.30 (1.28–4.13)	0.006
**PBC**				
Age, years old	1.01 (0.94–1.07)	0.960		
Male	0.83 (0.09–7.03)	0.861	0.56 (0.96–3.26)	0.509
BMI, kg/m^2^ ≥ BMI 25	0.90 (0.74–1.09) 0.73 (0.14–3.80)	0.286 0.697		
	
ALT, log IU/L **≥** UNL x 5	0.57 (0.22–1.49) -1.80 (-5.26–1.65)	0.245 0.299		
	
Bilirubin, mg/dL	2.24 (0.12–43.06)	0.583		
GGT, log IU/L	1.18 (0.65–2.12)	0.574		
ALP, log IU/L	1.58 (0.54–4.57)	0.392	1.14 (0.48–2.73)	0.763
Steatosis	0.79 (0.06–10.06)	0.852		
Activity grade	5.59 (1.91–16.42)	0.002	2.19 (0.73–6.56)	0.156
Fibrosis grade	5.11 (2.60–10.05)	<0.001	4.04 (1.82–8.96)	0.001

ElastPQ, elastography point quantification; CI, confidence interval; AILD, autoimmune liver disease; AIH, autoimmune hepatitis; BMI, body max index; ALT, alanine aminotransferase; UNL, upper normal limit; PBC, primary biliary cholangitis; GGT, gamma-glutamyl transpeptidase; ALP, alkaline phosphatase

### Comparison of ElastPQ with serum fibrosis markers

**Fig 2:**
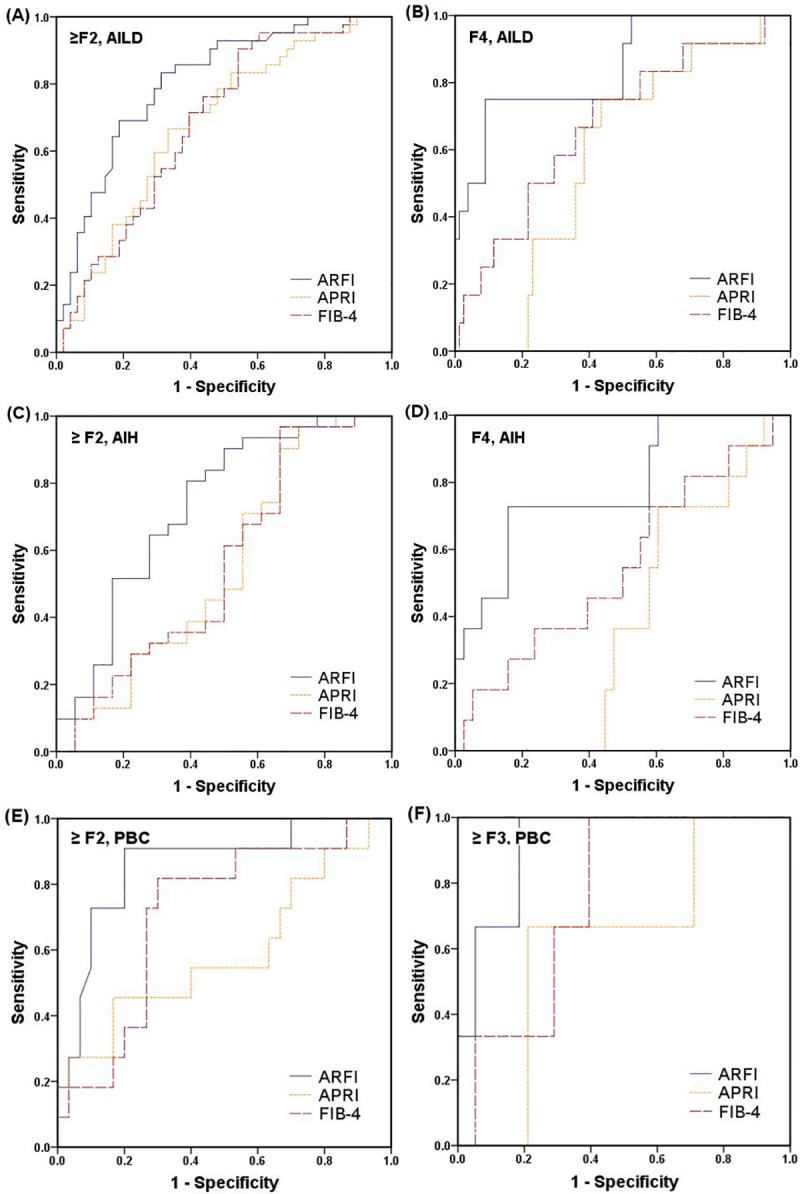
Receiver operating characteristic (ROC) curves of elastography point quantification (ElastPQ) and serum fibrosis markers for assessing significant hepatic fibrosis (≥ F2) and cirrhosis (F4) in autoimmune liver disease (A, B), autoimmune hepatitis (C, D), and significant hepatic fibrosis (≥ F2) and advanced fibrosis (≥ F3) in primary biliary cholangitis (E, F). ElastPQ has shown better diagnostic performance for assessing hepatic fibrosis than serum fibrosis markers, aspartate aminotransferase to platelet ratio index (APRI) and fibrosis-4 (FIB-4). APRI = (aspartate aminotransferase [AST] [IU/L] / upper normal limit of AST [IU/L]) / platelets [10^3^/mm^3^] FIB-4 = (age [years] × AST [IU/L]) / (platelets [10^3^/mm^3^] × alanine aminotransferase [ALT] [IU/L]^1/2^).

In patients with AIH and PBC, the AUC value of ElastPQ for predicting F4 fibrosis (0.81, 95% CI 0.65–0.96) was significantly higher than those of APRI (0.58, 95% CI 0.43–0.73) or FIB-4 (0.68, 95% CI 0.51–0.85); however, it did not show a statistically significant superiority for detecting ≥ F2 fibrosis (Table 4 and Fig 2). Among AIH patients, AUC of ElastPQ in predicting liver cirrhosis (F4, 0.75, 95% CI 0.60–0.94) was significantly higher than those of APRI (0.38, 95% CI 0.22–0.54, *P*<0.001) or FIB-4 (0.55, 95% CI 0.35–0.75, *P* = 0.012). For detecting significant hepatic fibrosis (≥ F2) in AIH, AUC of ElastPQ (0.70, 95% CI 0.54–0.87) tended to be higher than those of APRI (0.55, 95% CI 0.37–0.73, *P* = 0.074) or FIB-4 (0.56, 95% CI 0.37–0.73, *P* = 0.054) with a borderline statistical significance. Among PBC patients, AUC of ElastPQ for detecting significant fibrosis (≥ F2, 0.81, 95% CI 0.66–0.95) did not show a statistical difference compared with those of APRI (0.59, 95% CI 0.37–0.82) or FIB-4 (0.71, 95% CI 0.53–0.89), which also showed a borderline significant trend, as shown in Table 4.

**Table 4:** Comparison of AUC ElastPQ with APRI and FIB-4 to determine hepatic fibrosis.

Fibrosis stage	AUC value			
	ElastPQ	APRI	FIB-4	P value (vs ElastPQ) APRI / FIB-4
**Total AILD**				
≥ F2	0.77(0.67–0.86)	0.68 (0.57–0.79)	0.69 (0.58–0.80)	0.154 / 0.138
≥ F3	0.81(0.71–0.91)	0.72 (0.60–0.83)	0.74 (0.63–0.86)	0.198 / 0.260
F4	0.81(0.65–0.96)	0.58 (0.43–0.73)	0.68 (0.51–0.85)	0.008 / 0.028
**AIH**				
≥ F2	0.70 (0.54–0.87)	0.55 (0.37–0.73)	0.56 (0.37–0.74)	0.074 / 0.054
≥ F3	0.76 (0.62–0.89)	0.59 (0.43–0.75)	0.66 (0.50–0.81)	0.061 / 0.175
F4	0.75 (0.60–0.94)	0.38 (0.22–0.54)	0.55 (0.35–0.75)	<0.001 / 0.012
**PBC**				
≥ F2	0.81 (0.66–0.95)	0.59 (0.37–0.82)	0.71 (0.53–0.89)	0.088 / 0.294
≥ F3	0.91 (0.78–0.99)	0.62 (0.26–0.97)	0.75 (0.53–0.97)	0.136 / 0.113

AUC, area under the receiver-operator-characteristic curve; ElastPQ, elastography point quantification; APRI, AST to platelet ratio index; FIB-4, fibrosis-4; vs, versus; AILD, autoimmune liver disease; AIH, autoimmune hepatitis; PBC, primary biliary cholangitis

## Discussion

In the present study, the diagnostic performance of ElastPQ for assessing hepatic fibrosis was retrospectively analyzed in 90 patients with AILD, including 49 patients with AIH and 41 patients with PBC. The results of our study demonstrated a significant positive correlation between the median ElastPQ values and the stages of hepatic fibrosis in patients with AILD. The diagnostic performance of ElastPQ for assessing liver cirrhosis (F4) was significantly better than that of serum biochemical markers (APRI and FIB-4). After adjusting for the fibrosis grade in AILD patients, histological activity, steatosis, and BMI were not significant contributing factors for ElastPQ.

The overall diagnostic performance of pSWE for assessing hepatic fibrosis in this study was slightly lower than that in previous meta-analysis, which included a total of 518 patients with CLD from eight studies [24]. AUC in the prediction of significant fibrosis (≥ F2) and cirrhosis (F4) in patients with CLD was 0.87 (95% CI 0.83–0.92) and 0.93 (95% CI 0.89–0.97), respectively. Nonetheless, most patients enrolled in previous studies had viral hepatitis or steatohepatitis [9, 35]. The only other previous study with AIH patients showed a mean velocity of pSWE for significant fibrosis (F2-4, 2.28 ± 0.68 m/s) to be higher than those without fibrosis (F0-1, 1.20 ± 0.24, *P* = 0.002). However, there was no description of diagnostic accuracy due to small sample size (n = 15) [36]. For PBC patients, a previous study using pSWE to measure hepatic fibrosis showed a very high diagnostic accuracy (AUC 0.852 in child class A patients); however, the cut-off value was not reported [37]. Hence, to the best of our knowledge, the present study is the first to evaluate the diagnostic performance of ElastPQ in assessing hepatic fibrosis in patients with AIH and PBC, providing a comparison with APRI and FIB-4.

The standard reference of pSWE for predicting the stage of hepatic fibrosis may be affected by etiology and each specific equipment model [16, 38]. In patients with chronic hepatitis B, Ma *et al*. presented the optimal cut-off values for predicting significant fibrosis (≥ F2, 6.99 kPa) and cirrhosis (F4, 9.19 kPa), which were comparable to our study [39]. However, Ferraioli *et al*. defined higher cut-off values 7.3 and 13.3 kPa for predicting significant fibrosis (≥ F2) and cirrhosis (F4), respectively in chronic hepatitis C patients [40]. These values were higher than those reported in the present study. In another study for AILD by Righi *et al*., confined to mild fibrosis (≥ F1, 4.69 kPa), showed similar cut-off values with the present study (≥ F1, 5.35 kPa) [41]. A larger scale studies are needed to better define the standard reference and cut-off values for assessing hepatic fibrosis in patients with AILD.

Although there were limited studies on AILD patients, it has been revealed that the diagnostic performance and clinical utility of SWE other than TE were similar to that of TE in CLD mostly with viral hepatitis patients [42–46]. However, the method for generating the shear wave is different between the focused ultrasound beams in SWE other than TE and the external mechanical vibrator in TE [47, 48]. The mechanical impulse in TE may easily be affected by the narrow intercostal space, fatty tissue, ascites, and the natures of peripheral hepatic parenchyma, which can directly interfere with the propagation of shear wave. Moreover, TE cannot visualize the hepatic parenchyma. These factors may affect the reproducibility and diagnostic accuracy of TE [15, 49]. SWE other than TE, on the other hand, has been shown to be less affected by these confounding factors, which is in line with our results [50, 51].

In our study, the role of necroinflammatory activity of hepatic parenchyma on ElastPQ value showed mixed results. It was identified as a significant independent contributing factor for ElastPQ on multivariate analysis in the total AILD group, including both AIH and PBC. However, when analyzed separately, it was not considered as an independent significant contributing factor for ElastPQ (Table 3). Similar to our finding, a previous study reported that the elasticity and viscosity of hepatic parenchyma–as measured by elastographic imaging techniques–are mainly determined by the degree of hepatic fibrosis and less by the inflammatory activity or steatosis [52]. Although histologic activity was a significant factor affecting the ElastPQ value, its effect was attenuated in multivariable analysis due to the strong effect of fibrosis grade.

Liver inflammation indicated by high level of AST and/or ALT has been considered as the major potential confounding factor for liver stiffness measurement with ElastPQ [16]. Although it was not the independent determinant of ElastPQ value in our study (Table 3), ElastPQ showed to have better diagnostic performance especially in AIH, according to the additional analysis performed that excluded patients with ALT elevation of greater than 5 times the normal limits (S2 Table). Notably, the mean ALT level of our study population was higher than that of other studies with chronic hepatitis B and/or C patients [40, 53] because AIH is usually diagnosed at the acute hepatitis period in clinical practice [54]. This may worsen the diagnostic performance of ElastPQ in AIH.

In the present study, APRI and FIB-4 showed lower diagnostic accuracy for detecting hepatic fibrosis compared with previous studies [55, 56]. While hepatic fibrosis is a relatively steady state, the serum transaminases can be easily changed by hepatocellular injury in CLD patients, especially in AILD. Therefore, the serum biochemical markers determined by serum transaminases may not reflect the actual state of hepatic fibrosis in patients with AILD. A recent study showed that APRI and FIB-4 were unable to identify the majority (81–89%) of chronic hepatitis B patients with advanced fibrosis or cirrhosis, resulting in a miscalculation of 71% of patients without fibrosis as having clinically significant fibrosis [57]. This indicates that the calculated scores can be lower than the suggested cut-off values in clinically stable patients with advanced fibrosis, and can be higher in patients with active hepatitis even without hepatic fibrosis. ElastPQ, which has been shown to not be influenced by serum transaminase in this study, may provide better diagnostic performance than APRI and FIB-4.

The present study suggests a broad application of ElastPQ on AILD patients in assessing the stages of hepatic fibrosis. However, there are several limitations to consider when interpreting our findings. Some results of our study were not statistically significant due to the small number of the subjects in each fibrosis stage. Moreover, a separate validation set was not assigned. In particular, only a small number of PBC subjects with advanced fibrosis (2 with F3, 1 with F4) was enrolled; hence, the cut-off value for cirrhosis was not determined. Owing to ethical issues, liver biopsy was not performed in PBC patients with advanced fibrosis who could be easily diagnosed in the clinical setting. To create a definite standardized reference for assessing hepatic fibrosis in patients with AILD, there should be additional validating studies that evaluate ElastPQ in a large population study. Secondly, the diagnostic performance of ElastPQ was compared with noninvasive serum fibrosis markers in the present study, but not with TE, which is a more broadly-studied modality. Because this study was retrospective its design, physicians did not need to perform both TE and ElastPQ simultaneously in clinical practice. Thus, a comparative study of diagnostic performance between ElastPQ and TE is needed in the future.

In conclusion, the diagnostic performance of ElastPQ in patients with AIH and PBC for assessing hepatic fibrosis appears to be better than serum non-invasive markers, and histological activity, steatosis, and BMI were not independent contributing factors for ElastPQ. ElastPQ can be a useful non-invasive pSWE method for assessing hepatic fibrosis in patients with AILD.

## Supporting information

S1 File Dataset used in the present study. (XLSX)

S1 Table Multivariable analysis of the factors predicting ARFI value. (DOCX)

S2 Table Diagnostic performance of ElastPQ in AIH except high ALT (> 5 times ULN). (DOCX)
