# Supplementary material for: Diagnostic performance of a point shear wave elastography (pSWE) for hepatic fibrosis in patients with autoimmune liver disease
Source: PLoS One. 2019 Mar 11;14(3):e0212771. doi: 10.1371/journal.pone.0212771 (PMC6411150; doi:10.1371/journal.pone.0212771)
Supplement: S1 Table — (DOCX) [file pone.0212771.s002.docx]

**S1 Table. Adjusted AUC of ElastPQ**^®^ **for hepatic fibrosis stage**

| Fibrosis stage | AIH | PBC | Total AILD |
| --- | --- | --- | --- |
| ≥ F2 |  |  |  |
| Observed AUC | 0.70 (0.56-0.83) | 0.81 (0.65-0.91) | 0.77 (0.67-0.85) |
| Adjusted AUC | 0.68 | 0.90 | 0.80 |
| ≥ F3 |  |  |  |
| Observed AUC | 0.76 (0.62-0.87) | 0.91 (0.78-0.98) | 0.81 (0.71-0.88) |
| Adjusted AUC | 0.75 | 0.93 | 0.80 |
| F4 |  |  |  |
| Observed AUC | 0.75 (0.61-0.87) |  | 0.81 (0.71-0.89) |
| Adjusted AUC | 0.75 |  | 0.78 |

AUC, area under the receiver-operator-characteristic curve; ElastPQ^®^, elastography point quantification; AIH, autoimmune hepatitis; PBC, primary biliary cholangitis; AILD, autoimmune liver disease
