# Supplementary material for: Diagnostic performance of a point shear wave elastography (pSWE) for hepatic fibrosis in patients with autoimmune liver disease
Source: PLoS One. 2019 Mar 11;14(3):e0212771. doi: 10.1371/journal.pone.0212771 (PMC6411150; doi:10.1371/journal.pone.0212771)
Supplement: S2 Table — (DOCX) [file pone.0212771.s003.docx]

**S2 Table. Diagnostic performance of ElastPQ**^®^ **for hepatic fibrosis stage in autoimmune liver disease patients except high ALT ( > 5 times ULN)**

|  | Cut-off (kPa) | AUC | Sensitivity (%) | Specificity (%) | P value |
| --- | --- | --- | --- | --- | --- |
| **AILD (n=67)** |  |  |  |  |  |
| ≥ F2 | 5.70 | 0.81 | 76.7 | 73.0 | <0.001 |
| ≥ F3 | 6.40 | 0.86 | 75.0 | 80.4 | <0.001 |
| F4 | 9.28 | 0.83 | 58.3 | 96.4 | <0.001 |
| **AIH (n=28)** |  |  |  |  |  |
| ≥ F2 | 4.47 | 0.82 | 94.7 | 55.6 | <0.001 |
| ≥ F3 | 7.11 | 0.84 | 77.0 | 93.3 | <0.001 |
| F4 | 9.28 | 0.80 | 54.6 | 94.1 | 0.002 |
| **PBC (n=39)** |  |  |  |  |  |
| ≥ F2 | 5.56 | 0.80 | 81.8 | 71.4 | <0.001 |
| ≥ F3 | 6.04 | 0.91 | 100 | 80.6 | <0.001 |

ElastPQ^®^, elastography point quantification; AILD, autoimmune liver disease; AIH, autoimmune hepatitis; PBC, primary biliary cholangitis; ALT, alanine aminotransferase; ULN, upper limit of normal; kPa, kilopascal; AUC, area under the receiver-operator-characteristic curve
